# Accidental Origin of the Mpemba Effect in Freezing: Reversals in the Freezing Order Are Stochastic—Experimentation and Theory

**DOI:** 10.3390/molecules31213954

**Published:** 2026-11-02

**Authors:** Andrei A. Klimov, Alexei V. Finkelstein

**Affiliations:** Institute of Protein Research, Russian Academy of Sciences, Pushchino 142290, Moscow Region, Russia

**Keywords:** Mpemba effect, first-order phase transition, water super-cooling, ice nucleation, ice-binding surfaces, temperature, single-event phenomenon, accidental phenomenon, freezing, uncertainty in freezing time

## Abstract

The “Mpemba effect” is the name given to the assertion that hot water may freeze faster than cool water. This counterintuitive statement seemingly breaches fundamental laws of physical chemistry; however, numerous experiments with classical and quantum systems have apparently confirmed it, sparking debate in prominent scientific journals. Here, we report on water freezing experiments conducted under carefully controlled conditions; they show that the Mpemba effect in water freezing exists as a stochastic, that is, accidental, rather than a deterministic phenomenon, which—as the theory shows—is due to the fundamental uncertainty in the onset time of a first-order phase transition (like freezing) close to the temperature of nucleation of this transition. This uncertainty results in a huge scattering of freezing onset times (both for initially cool and initially hot water), so that, close to the temperature of ice nucleation, initially hot water *sometimes* freezes faster and *sometimes* slower than initially cool water (whereas at lower temperatures, this *almost never* happens). We anticipate our essay to be a starting point for reconsidering the famous Mpemba paradox in freezing water and other systems undergoing first-order phase transitions.

## 1. Introduction

Nearly 60 years ago, the surprising claim that “the water that starts hotter may freeze first” was dubbed the Mpemba effect [1]; the classical Mpemba effect implies that, under certain conditions, any (or almost any) vial of hot water freezes before any (or almost any) vial of cool water. Similar counterintuitive phenomena, seemingly defying basic physics and chemistry, have since been observed in liquids, polymers, clathrate hydrates, colloids, and even quantum systems [2,3,4,5,6,7,8,9,10,11,12,13,14,15] and discussed in many theoretical studies [8,9,11,13,16,17,18,19,20,21,22,23,24,25,26,27,28,29,30,31,32,33]. This reflects burgeoning research on this effect, which is of interest to chemists in various areas, highlighting that the underlying physicochemical mechanisms remain elusive [31], and that even the seemingly simple process of water freezing is not fully understood.

Our water freezing experiments demonstrate that the Mpemba effect in freezing occurs *stochastically*, accidentally, and almost exclusively when the freezer temperature is very close to that of ice nucleation. As the theory shows, all this is due to the stochastic uncertainty in the timing of ice nucleation, this first-order phase transition, and does not require any hypothesis about any special freezing mechanism or “water memory”.

## 2. Results

### 2.1. Experiment

To clarify the basis of the in-water Mpemba paradox, we conducted experiments replicating previously reported studies on water freezing [1,2,3,4,7,15] under carefully controlled conditions (see Section 4 and Supplementary Materials S1).

Consistent with seminal studies [1,3], we focused [34] on the *onset* of freezing rather than on the complete freezing of ice or the cooling of water to 0 °C. The latter has been suggested by careful experiments on water cooling to 0 °C without freezing [4], which show that hot water *does not* cool faster than cold water. This allowed us to neglect water evaporation [35,36] (especially since in our and some other [1,2,3,4] experiments, the water samples were kept in closed vials) and convection [37,38] as causes of the Mpemba effect and focus on the phase transition that apparently drives this effect in various systems, including milk [1], water [1,2,15], clathrate hydrates [5], and polymers [6].

**Figure 1:**
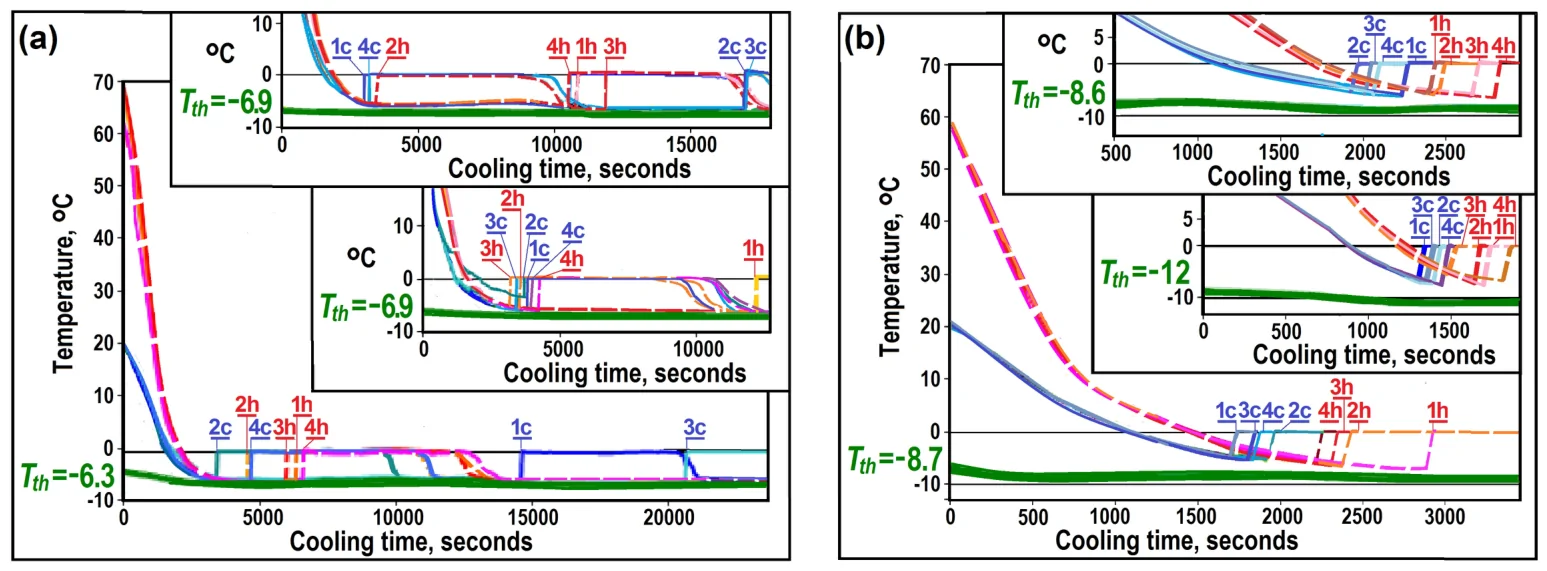
Dynamics of water cooling and freezing at thermostat temperatures *T_th_* above −7 °C (**a**) and below −8 °C (**b**). In each experiment, the four “cool” water samples started at +20 °C (1c–4c, thin blue lines), whereas the four “hot” ones started at +70 °C (1h–4h, thin red dashed lines). Each line tracks the temperature evolution of one sample. Sharp temperature increases (marked by 1c–4c, 1h–4h) indicate the onset of freezing, causing a sharp release of latent heat [39]. Bold green lines represent the measured freezer temperatures *T_th_*.

Figure 1 shows the dynamics of cooling and freezing water samples (placed in closed vials) at different thermostat temperatures *T_th_*.

In each experiment, the four “cool” water samples started at +20 °C, whereas the four “hot” ones started at +70 °C; all of these were positioned in the freezer simultaneously. When cooling, all samples of the initially hot water reached 0 °C approximately 500 s later than all samples of the initially cool water (which agrees with [4]; with further cooling at *T_th_* > −7 °C, some samples of the initially hot water froze earlier, and some later, than the samples of the initially cool water, but at *T_th_* < −8 °C all the samples of the initially hot water froze later than all the samples of the initially cool water.

The main panel of Figure 1a illustrates the full temperature evolution in one experiment with eight water samples at a thermostat temperature *T_th_* above −7 °C, while the main panel of Figure 1b illustrates the full temperature evolution at a thermostat temperature *T_th_* below −8 °C. The insets in Figure 1a,b show the low-temperature dynamics of the behavior of water samples in two other experiments (among many others; see Table 1a,b) conducted at thermostat temperatures above −7 °C (a) and below −8 °C (b).

Table 1a,b present detailed data on the cooling and freezing of all initially hot and cool water samples at different thermostat temperatures *T_th_*.

At the freezer temperature *T_th_* ≥ −7.2 °C (Figure 1a and Table 1a), one can see a *large* variation in freezing onset times indicated by sharp temperature increases [39]. For the “initially cool” (+20 °C) and “initially hot” (+70 °C) water samples, the ranges of the onset times *do* overlap, such that at this relatively high *T_th_* (−7.2 °C and higher), the Mpemba effect *is* observed, *but* it is *stochastic*—it is observed for *some* pairs of samples but *not* for the others.

After each experiment (for *T_th_* ≥ −7.2 °C, we conducted eight independent experiments with four “initially hot” and four “ initially cool” water samples; see Table 1a), we performed 16 comparisons of these four “hot” and four “cool” samples. The comparisons were not entirely independent, as in each experiment, we dealt with only eight independent numbers; however, the ratio of the sums of the resulting numbers—shown in the last two columns—provides a simple, general overview of the relationship between the “hot freezes before cool” and the “cool freezes before hot” cases. In total, we had 4 × 4 × 8 = 128 comparisons. The Mpemba effect (the “hot” sample freezes first) occurred in 41% of the cases. For example, the main panel of Figure 1a shows that “hot” samples 1h–4h began to freeze *earlier* than the “cool” samples 1c and 3c but *later* than the “cool” sample 2c.

Conversely, at *T_th_* < −8 °C (Figure 1b and Table 1b), one can see a *small* variation in the freezing onset times. For the +20 °C and +70 °C samples, the onset times *practically do not* overlap, so the initially cool water *virtually always* (in 170 cases out of 176) freezes first (and below −10 °C, initially cool water *always* freezes first); thus, at the low *T_th_* (below −8 °C), the Mpemba effect is practically *not* observed. This is proven by 4 × 4 × 11 = 176 comparisons of four “initially hot” and four “ initially cool” water samples (the caveat made earlier—that these comparisons are not entirely independent—does not alter this general conclusion).

Thus, our water freezing experiments highlight (*i*) the stochastic nature of the onset of freezing, especially at *T_th_* > −7 °C; and (*ii*) the significant role of the “not very cold” (−7.2 °C and higher) freezer temperature *T_th_* in inducing this *stochastic* Mpemba effect.

Previous experimental papers have sometimes hinted at the stochastic, accidental nature of the Mpemba effect, using cautious phrases such as “the water that starts hotter *may* freeze first” [1] or “hot water *may well* begin to freeze quicker than cold” [13], but no comprehensive investigation into this critical aspect of the Mpemba effect related to ice nucleation has been reported.

Here, we will outline and try to explain the conditions under which the stochasticity in ice nucleation time *sometimes* leads to the observation of the Mpemba effect.

It should be noted that the statement about the stochasticity of the Mpemba effect applies only to events involving first-order phase transitions (see below). Systems *without* these transitions, such as colloids [8,11], some quantum [13,14,29,30,32], and mechanical [40,41] systems, can exhibit a deterministic rather than stochastic Mpemba effect.

### 2.2. Theory

The freezer temperature *T_th_* impacts both freezing and cooling rates.

The latter, according to Newton’s law of thermal conductivity, can be described as

*dT_t_*/*dt* = −κ · (*T_t_* − *T_th_*); (1)

where *κ* is the thermal conductivity coefficient attributed to the surroundings of the water sample (having temperature *T_t_*), and *t* is time. This equation implies that *d*[*ln*(*T_t_* − *T*_th_)] = −*κ* · *dt*; so,

*T_t_* − *T_th_* = (*T*_1_ − *T_th_*) *exp*(−κ · *t*), (2)

where *T*_1_ > *T_th_* represents the temperature of the sample at the initial moment (*t*_1_ = 0) of cooling. Thus, the time required for the sample to cool from *T*_1_ to *T_t_* is

(3) $$t_{\mathrm{th}}\left(T_{1},T_{t}\right)=\frac{1}{\kappa }\ln\frac{T_{1}-T_{\mathrm{th}}}{T_{t}-T_{\mathrm{th}}};$$

where *κ*~0.002 s^−1^ corresponds to cooling by a freezer set at *T_th_* of about −5 ÷ −15 °C (as in our experiments and in seminal works [1,2], where cooling from *T*_1_ ≈ +80 °C to *T_t_* ≈ +20 °C took about 10 min).

This *κ*~0.002 s^−1^ estimate will be used throughout our study for both “initially hot” and “initially cool” water samples without fitting to each cooling trace, since the exact value of *κ* is not crucial for understanding the nature of the Mpemba effect.

Water freezing can start only when *T_t_* is sufficiently [2,39,42] below *T*_0_ ≡ 273 K, i.e., 0 °C.

According to the conventional Gibbs transition state theory for first-order phase transitions [39,43,44,45] (like water freezing), the time to the appearance of an ice nucleus (corresponding to the most unstable, i.e., transition state during ice formation) around one given H_2_O molecule in still pure water can be estimated as

(4) $$t_{T}^{\left(1\right)}\sim \tau \cdot \exp\left(\frac{{+G}_{\mathrm{nucl}}^{\#}(T)}{k_{B}T}\right).$$

Here, $G_{\mathrm{nucl}}^{\#}(T)$ is the free energy of the ice nucleus at temperature *T < T*_0_; *k_B_* is the Boltzmann constant; and *τ* is the typical time for one H_2_O molecule to attach to ice. The value of *τ* is no less than *τ*_0_~10^−12^ s (the typical time [45] of thermal vibrations at 0 °C); in fact, *τ* is estimated (see [45], chs. 3.2 and 8.2) from the difference between the rates of H_2_O molecule attachment to and detachment from ice. The final estimate of *τ* also includes the “Zeldovich factor”, i.e., the number of steps spent to cross the top of the activating barrier, and the number of possible parallel nucleus growth pathways [42]. With only the dominant terms considered [42,45], *τ*~10^−7^ s at “normal” sub-zero temperatures (−1 °C to−20 °C). This value of *τ* depends on the temperature but much less so than $\exp\left(\frac{{+G}_{\mathrm{nucl}}^{\#}(T)}{k_{B}T}\right)$, which rapidly tends toward infinity as the temperature *T* approaches 0 °C (so that the water freezing temperature must be strictly below 0 °C for kinetic reasons [39,42]).

The free energy $G_{\mathrm{nucl}}^{\#}$ is very large for a three-dimensional (3D) ice nucleus in bulk water [42], where ice, for kinetic reasons, arises only below −35 °C [46].

However, the two-dimensional (2D) ice nucleus on an ice-binding surface has a smaller $G_{\mathrm{nucl}}^{\#}(T)$; thus, ice can arise [42] on an ice-binding border of water at negative temperatures closer to 0 °C. Then $G_{\mathrm{nucl}}^{\#}(T)$ can be estimated as $\approx 4B_{2}\left(\frac{B_{2}}{-∆\mu (T)}\right)$ [42,45], where Δ*μ*(*T*) is the chemical potential of H_2_O in ice minus that in water (so that ∆*μ*(*T*_0_) = 0, and ∆*μ*(*T*) < 0 at *T < T*_0_, where $\frac{∆\mu (T)}{k_{B}T}\approx \frac{T-T_{0}}{100{}^{\circ}}$; see [45], with data taken from [47]), and *B*_2_ is the additional free energy of an ice molecule at the ice/water interface, with *B*_3_ ≈ 0.85 *k_B_T*_0_ for a 3D ice/water interface [48] (see also [45], with data from [47]); this estimate seems to remain more or less valid for the perimeter of a 2D ice layer (i.e., *B*_2_ ≈ *k_B_T*_0_) [49]. With these ∆*μ*(*T*) and *B*_2_ estimates, as well as *T* < *T*_0_ (but close to *T*_0_), we have [42,49,50]

(5) $$\frac{G_{\mathrm{nucl}}^{\#}(T)}{k_{B}T}=\frac{T_{*}}{T_{0}-T}$$

where T_*_ ≈ 400° at *B*_2_ ≈ *k_B_T*_0_. This estimate captures the main effect: an enormous growth of $G_{\mathrm{nucl}}^{\#}(T)$ when *T* approaches the ice-and-water coexistence temperature of 0 °C.

Since, at a given temperature *T* < *T*_0_, the time to the appearance of an ice nucleus around *one* given on-border H_2_O molecule is determined by Equations (4) and (5), the time to appearance of an ice nucleus around *one of the N* on-border H_2_O molecules existing on the surface of the vessel (where, in the absence of dust, ice can only appear at sub-zero temperatures) can be estimated as

(6) $$t_{T}^{\left(N\right)}\sim \frac{\tau }{N}\cdot \exp\left(\frac{T_{*}}{T_{0}-T}\right).$$

With the number of on-border waters *N* ~ 10^15^ in a ~1 mL test tube (since a single water molecule with a diameter of ≈3 Å covers ≈10^−15^ cm^2^ of the test tube wall, and different water molecules can be roughly considered as independent potential nucleation sites) and (see above) *τ*~10^−7^ s approximately, one can see from this equation that the value of $t_{T}^{\left(N\right)}$ theoretically amounts to minutes at the water temperature *T* = *T*_0_ − 7 °C, days at *T* = *T*_0_ − 6 °C, and ages at *T* = *T*_0_ − 5°C.

Thus, the freezing of pure water is only possible at water temperatures *T* below *T_freeze_* corresponding to ≈−6 °C. It is worth noting that changing the values of *N* and *τ* by an order of magnitude each leads to roughly the same estimates of $t_{T}^{\left(N\right)}$ (minutes, days, and ages at ≈−7, ≈−6, and ≈−5 °C) when the fitting parameter T_*_ is varied by only 10%.

The probability of appearance of an ice nucleus around one given on-border H_2_O molecule during the above-specified time *τ* at temperature *T* < *T*_0_ is $p_{T<T_{0},\tau }^{\left(+1\right)}\equiv \frac{\tau }{t_{T}^{\left(1\right)}}=\exp\left(\frac{{-T}_{*}}{T_{0}-T}\right)$, while $p_{T>T_{0},\tau }^{\left(+1\right)}=0$ at *T* ≥ *T*_0_. Thus,

(7) $$p_{T<T_{0},\mathrm{dt}}^{\left(+1\right)}=\frac{\mathrm{dt}}{\tau }\cdot \exp\left(\frac{-T_{*}}{T_{0}-T}\right)$$

is the probability of the nucleus appearing around a given on-border H_2_O molecule during a short time *dt*, and $p_{T,\mathrm{dt}}^{\left(-1\right)}=1-p_{T,\mathrm{dt}}^{\left(+1\right)}$ is the probability that a nucleus *does not* appear during this time *dt* around this on-border water molecule. So, the value ${p_{T<T_{0},\mathrm{dt}}^{\left(-N\right)}=\left[1-p_{T<T_{0},\mathrm{dt}}^{\left(+1\right)}\right]}^{N}\approx \exp\left(-Np_{T<T_{0},\mathrm{dt}}^{\left(+1\right)}\right)=\exp\left[-\mathrm{dt}\frac{N}{\tau }\exp\left(\frac{{-T}_{*}}{T_{0}-T}\right)\right]$ is the probability that, at *T* < *T*_0_ in a system of *N* on-border waters, *not a single* nucleus arises during the time *dt*. Then, $\prod_{i=1}^{m}p_{T_{i}<T_{0},dt_{i}}^{\left(-N\right)}=$ $\exp\left[-\sum_{i=1}^{m}dt_{i}\frac{N}{\tau }\exp\left(\frac{{-T}_{*}}{T_{0}-T_{i}}\right)\right]$ is the probability that *not a single* nucleus arises in this system during the time interval *dt*_1_+…+*dt_m_* when all temperatures *T*_1_,…,*T_m_* are below *T*_0_.

A freezer set at *T_th_* < *T*_0_ needs (see Equation (3)) time ${t_{0}=t}_{\mathrm{th}}(T_{1},T_{0})$ for cooling water from *T*_1_ to *T*_0_, only after which ice can arise. At times above *t*_0_ (when *T_t_* falls below *T*_0_),

(8) $$S_{t^{*}>t_{0}}^{-}=\exp\left[-\int_{t_{0}}^{t^{*}}\mathrm{dt}\frac{N}{\tau }\exp\left(\frac{{-T}_{*}}{T_{0}-T_{t}}\right)\right]$$

represents the probability that no nucleus arises among *N* of on-border waters during time *t*_0_ − t^*^ (note that $S_{{t\leq t}_{0}}^{-}\equiv 1$, and $S_{t^{*}{>t}_{0}}^{-}$ tends to zero at t^*^ → *∞* when all of *T_t_* < *T*_0_ at *t*_0_ ≤ *t* ≤ t*). Thus,

(9) $$S_{t^{*}>t_{0}}^{+}\equiv 1-S_{t^{*}>t_{0}}^{-}=1-\exp\left[-\int_{t_{0}}^{t^{*}}\mathrm{dt}\frac{N}{\tau }\exp\left(\frac{{-T}_{*}}{T_{0}-T_{t}}\right)\right]$$

is the probability that the first nucleus arises in the on-border water during the time interval *t*_0_ − t* (and $S_{{t\leq t}_{0}}^{+}=0$). So, the probability of ice nucleation during the short time interval t* − (t* + dt*) is $dt^{*}\left\{\frac{d}{dt^{*}}S_{t^{*}>t_{0}}^{+}\right\}$, which equals to $\left\{\exp\left[-\int_{t_{0}}^{t^{*}}\mathrm{dt}\frac{N}{\tau }\exp\left(\frac{{-T}_{*}}{T_{0}-T_{t}}\right)\right]\cdot \left[\frac{N}{\tau }\exp\left(\frac{{-T}_{*}}{T_{0}-T_{t^{*}}}\right)\right]\right\}\cdot dt^{*}$. Thus, at t^*^ > *t*_0_,

(10) $$P_{t^{*}-t_{0}}^{+}\equiv \frac{d}{dt^{*}}S_{t^{*}>t_{0}}^{+}=\exp\left[-\int_{t_{0}}^{t^{*}}\mathrm{dt}\frac{N}{\tau }\exp\left(\frac{{-T}_{*}}{T_{0}-T_{t}}\right)\right]\cdot \left[\frac{N}{\tau }\exp\left(\frac{{-T}_{*}}{T_{0}-T_{t}}\right)\right]$$

is the momentary probability of ice nucleation at t^*^ > *t*_0_ (note: $P_{t^{*}\leq t_{0}}^{+}$ = 0, $\int_{t_{0}}^{\infty }P_{t^{*}>t_{0}}^{+}dt^{*}$ = 1, while Equation (3) gives the time *t*_0_ = *t_th_*(*T*_1_,*T*_0_) needed to cool water to the temperature *T*_0_, as well as the time $t_{\mathrm{th}}\left(T_{1},T_{t^{*}}\right)$ needed to cool ice-free water to the temperature $T_{t^{*}}$).

**Figure 2:**
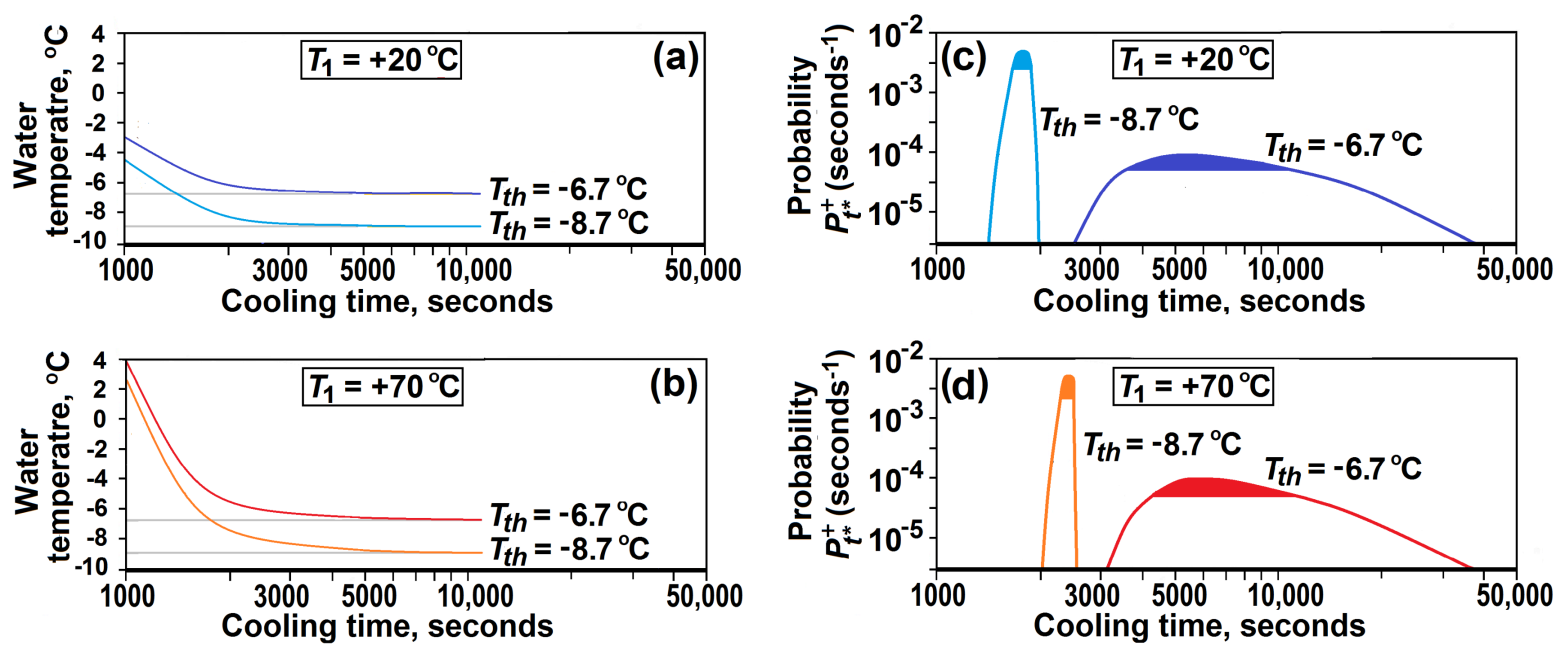
Computed (see Equation (2)) Newtonian cooling dynamics of water in the absence of freezing (**a**,**b**) and computed (using Equation (10)) probabilities $P_{t^{*}}^{+}$ of ice nucleation (**c**,**d**) as functions of the cooling time at different freezer temperatures *T_th_*. Cooling begins at initial temperatures *T*_1_ = +20 °C (**a**,**c**) and *T*_1_ = +70 °C (**b**,**d**). The only difference between freezing in (**c**,**d**) is the time delay of about 500 s, reflecting the additional time required to cool hot water from +70 °C to +20 °C. A comparison of the panels shows that the change in water temperature over the timescales required for ice nucleation is quite small.

Figure 2 shows the computed cooling dynamics of water and computed probabilities $P_{t^{*}}^{+}$ of ice nucleation as functions of the cooling time t^*^. These computations use Equations (2) and (10) with *N* = 10^15^, *τ* = 10^−7^ s, T_*_ = 400°, and *κ* = 0.002 *s*^−1^ for cooling from the initial temperatures *T*_1_ = +20 °C (panels 2(a,c)) and *T*_1_ = +70 °C (panels 2(b,d)) at various thermostat temperatures *T_th_*.

Thus, as illustrated by Figure 2 (in accordance with Figure 1 and the graphical Abstract) for both initial temperatures, the expected ranges of ice nucleation times are quite wide (>>1000 s, see caps on the tops of $P_{t^{*}}^{+}$ peaks) and overlap at freezer temperatures above −7 °C; however, at lower (below −8 °C and especially −10 °C) freezer temperatures, they are narrow (less than ≈500 s delay in the cooling of hot water) and do not overlap.

This theoretical result agrees with experimental observations across water samples cooled at different freezer temperatures (Figure 1a,b), supporting the conclusion that the “Mpemba effect” manifests itself—but only in a fraction of experiments—as a *stochastic* event, and almost exclusively at relatively high freezer temperatures *T_th_* above ≈−7 °C.

## 3. Discussion

Figure 1 and Table 1a,b clearly demonstrate the following striking features:

(i) Water (in a closed vial) freezes not at 0 °C but (for kinetic reasons [39,42,45,49,50]) only below ≈−6 °C, i.e., in a super-cooled state [2,39]—even then, it does not immediately start to freeze.
(ii) The typical time to the onset of water freezing during cooling is very large, with *T_th_* of about −6 ÷ −7 °C [2,39,45]; this time decreases significantly (approximately tenfold) with decreasing thermostat temperature *T_th_* from −6.3 ÷ −6.9 to −8.6 ÷ −8.7 °C, i.e., by only ≈ 2°—this accords to the above (Figure 2c,d) estimates.
(iii) At the freezer temperature *T_th_* above −7.2 °C, freezing occurs at water temperature *T_fr_* that is only slightly above this *T_th_*, typically by ≲0.5°, while at the lower temperature *T_th_*, corresponding to −8 ÷ −12 °C, freezing occurs at a water temperature *T_fr_* that is close to ≈−6.5 °C and thus noticeably above this low *T_th_* by about 2° ÷ 5°.
(iv) At the thermostat temperature *T_th_* corresponding to ≈ −8.6 °C and below, the scattering of the freezing onset times is relatively small; for all samples of both initially cool and initially hot water, this scattering is less than 500 s (that is, less than the typical delay in the cooling of initially hot water), whereas at the thermostat temperature *T_th_* above −7 °C, there is a huge (~10,000 s) scattering of freezing onset times, both for initially cool and initially hot water (which is far beyond the typical ≈500 s delay in cooling of initially hot water); as such, initially hot water *sometimes* freezes faster and *sometimes* slower than initially cool water, which also corresponds to what is seen in Figure 2.

To grasp why higher freezer temperatures *T_th_* result in wider freezing time ranges, while a lower *T_th_* yields narrower freezing time scattering, we can find an approximate solution to Equation (10) by simplifying the consideration of the cooling and freezing process by dividing it into two stages:

(a) *Cooling* when the temperature drops from *T*_1_ to *T_fr_*, which takes time *t*_c_ = *t_th_*(*T*_1_,*T_fr_*).
(b) *Freezing onset* at the constant temperature *T_fr_* < *T*_0_.

It should be noted that the purpose of this simplification is to provide a general understanding of the cooling and freezing process, rather than a precise description of its details. The approximation used is justified by the fact that the change in water temperature on the timescale at which ice formation begins is quite small (see Figure 2), and it allows for the use of deliberately approximate, rounded numerical parameters.

During cooling with a freezer set at the temperature *T_th_*, the most probable ice-nucleation temperature *T_fr_*(*T_th_*) corresponds to the minimum (by *T_fr_*) of the sum of the *cooling* time, *t_th_*(*T*_1_,*T_fr_*)—see Equation (3)—and the following (after reaching *T_fr_*) mean *freezing onset* time ${\langle t_{f}\rangle }_{T_{\mathrm{fr}}}=\int_{0}^{\infty }t\cdot P_{t}^{+}\mathrm{dt}$. Denoting the constant, at *T_t_* = *T_fr_* = *const*, value $\frac{N}{\tau }\exp\left(\frac{{-T}_{*}}{T_{0}-T_{\mathrm{fr}}}\right)$ as $A_{T_{\mathrm{fr}}}$, we obtain from Equation (10) $P_{t}^{+}=A_{T_{\mathrm{fr}}}\exp(-A_{T_{\mathrm{fr}}}t)$ at *t* > 0, expressed as

(11) $${\langle t_{f}\rangle }_{T_{\mathrm{fr}}}=\int_{0}^{\infty }t\cdot A_{T_{\mathrm{fr}}}\exp\left(-A_{T_{\mathrm{fr}}}t\right)\cdot \mathrm{dt}=\frac{1}{A_{T_{\mathrm{fr}}}}\equiv \frac{\tau }{N}\exp\left(\frac{T_{*}}{T_{0}-T_{\mathrm{fr}}}\right),$$

which drastically grows when *T_fr_* approaches *T*_0_.

The minimum (by *T_fr_*) of the sum $t_{\mathrm{th}}\left(T_{1},T_{\mathrm{fr}}\right)+{\langle t_{f}\rangle }_{T_{\mathrm{fr}}}$= $\frac{1}{\kappa }\ln\frac{T_{1}-T_{\mathrm{th}}}{T_{\mathrm{fr}\;}-T_{\mathrm{th}}}+\frac{\tau }{N}\exp\left(\frac{T_{*}}{T_{0}-{\;T}_{\mathrm{fr}}}\right)$ is achieved when

(12) [T0− Tfr/T*]2Tfr−Tth/T*=τNexpT*T0−Tfr11κ.

**Figure 3:**
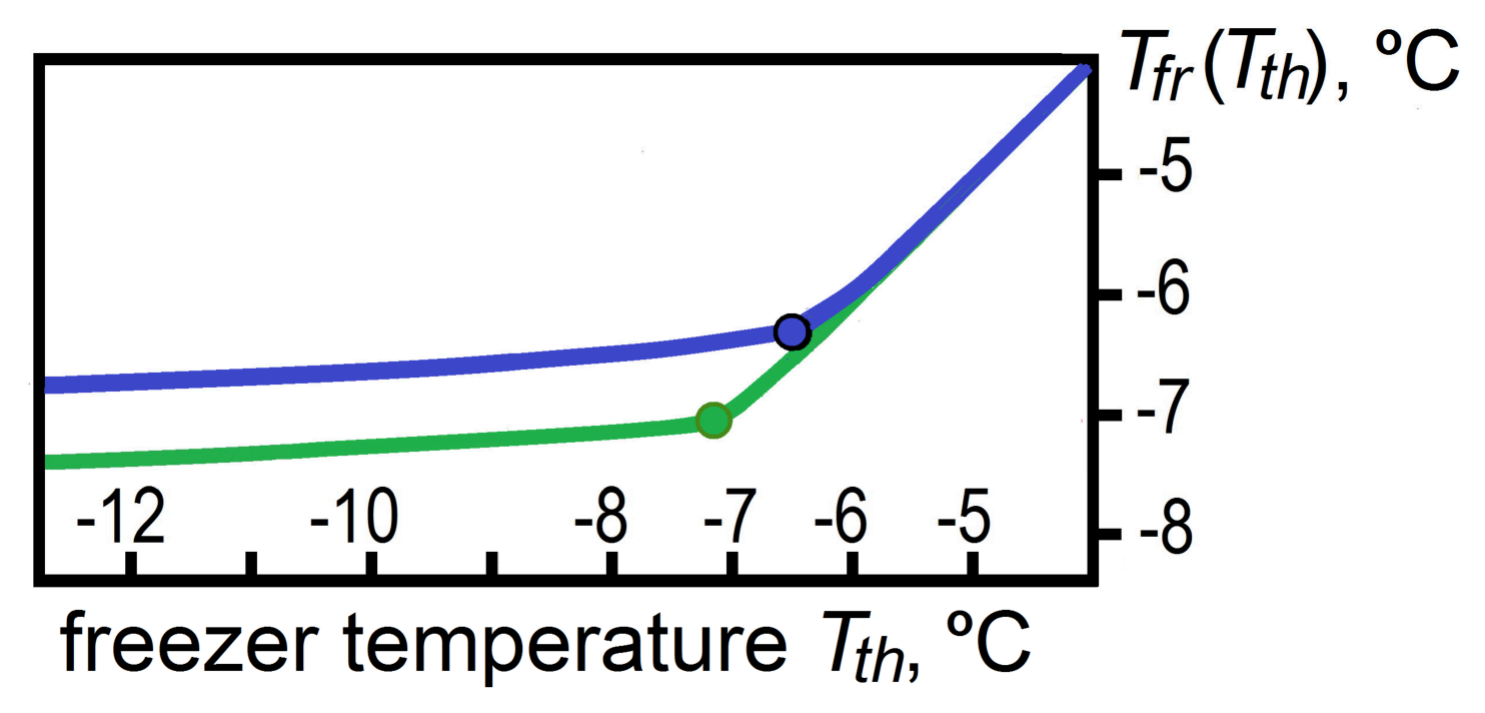
Theoretically expected from Equation (12) ice nucleation temperatures *T_fr_* as a function of the freezer temperature *T_th_*. Calculations were done using *N* = 10^15^, *τ* = 10^−7^ s, *κ* = 0.002 *s*^−1^, and T_*_ = 400° (green line); and *N* = 10^15^, *τ* = 10^−7^ s, *κ* = 0.0022 *s*^−1^, and T_*_ = 360° (blue line). To the left of the circles, the values of *T_fr_* differ significantly from the freezer temperatures *T_th_*; to the right of these circles, the values of *T_fr_* are very close to *T_th_*.

The solution of Equation (12) gives *T_fr_* as a function of *T_th_* (see Figure 3).

This solution has two regimes: (A) when $\frac{[{\left(T_{0}-T_{\mathrm{fr}}\right)/T_{*}]}^{2}}{\left(T_{\mathrm{fr}}-T_{\mathrm{th}}\right)/T_{*}}>1$, and (B) when $\frac{[{\left(T_{0}-T_{\mathrm{fr}}\right)/T_{*}]}^{2}}{\left(T_{\mathrm{fr}}-T_{\mathrm{th}}\right)/T_{*}}<1$, separated by a point where $\frac{[{\left(T_{0}-T_{\mathrm{fr}}\right)/T_{*}]}^{2}}{\left(T_{\mathrm{fr}}-T_{\mathrm{th}}\right)/T_{*}}=1=\frac{\kappa \tau }{N}\exp\left(\frac{T_{*}}{T_{0}-T_{\mathrm{fr}}}\right)$. At this point (marked by a green closed circle in Figure 3), $T_{\mathrm{fr}}-{T_{0}=-T}_{*}/\ln\left(\frac{N}{\kappa \tau }\right)$ and $T_{\mathrm{th}}=T_{\mathrm{fr}}{-T}_{*}/{\left[\ln\left(\frac{N}{\kappa \tau }\right)\right]}^{2}$.

The solution for Equation (12) has two regimes:

(A) When $\frac{[{\left(T_{0}-T_{\mathrm{fr}}\right)/T_{*}]}^{2}}{\left(T_{\mathrm{fr}}-T_{\mathrm{th}}\right)/T_{*}}>1$—this corresponds to the case where *T_fr_* is very close to *T_th_*, and the cooling and freezing time is determined primarily by the freezing onset time.
(B) When $\frac{[{\left(T_{0}-T_{\mathrm{fr}}\right)/T_{*}]}^{2}}{\left(T_{\mathrm{fr}}-T_{\mathrm{th}}\right)/T_{*}}<1$—in this case *T_fr_* is not close to *T_th_*, and the cooling and freezing time is determined primarily by the cooling.

These regimes are separated by a point (marked by a circle) where $\frac{[{\left(T_{0}-T_{\mathrm{fr}}\right)/T_{*}]}^{2}}{\left(T_{\mathrm{fr}}-T_{\mathrm{th}}\right)/T_{*}}=1=\frac{\kappa \tau }{N}\exp\left(\frac{T_{*}}{T_{0}-T_{\mathrm{fr}}}\right)$. At this point, $T_{\mathrm{fr}}={T_{0}-T}_{*}/\ln\left(\frac{N}{\kappa \tau }\right)$ and $T_{\mathrm{th}}=T_{\mathrm{fr}}{-T}_{*}/{\left[\ln\left(\frac{N}{\kappa \tau }\right)\right]}^{2}$.

With the above values of *N* = 10^15^, *τ* = 10^−7^ s, *κ* = 0.002 *s*^−1^, and T_*_ = 400°, one obtains *T_th_* corresponding to −7.16 °C, and *T_fr_*(*T_th_*) corresponding to −7.03 °C (see the green line in Figure 3). With numerical parameters that differ by 10% (*κ* = 0.0022 *s*^−1^ and T_*_ = 360°, while *N* and *τ* remain as specified above), we obtain *T_th_* corresponding to −6.45 °C and *T_fr_*(*T_th_*) to −6.34 °C (see the blue line in Figure 3). These resulting 10% changes in the values of *T_th_* and *T_fr_* have practically no effect as we shall see below on the explanation of why the onset times and their variabilities are immense at higher freezer temperatures and comparatively small at lower temperatures; however, the freezing onset temperatures *T_fr_*, calculated with *κ* = 0.0022 *s*^−1^ and T_*_ = 360°, seem to be closer to the experimentally measured values (see Figure 1 and Notes 2 to Table 1, parts(a) and (b)).

Having estimated the dependence of *T_fr_* on *T_th_*, we can use Equations (3) and (11) to find the estimate of *t*_c_(*T_th_*), i.e., of the *T*_1_ → *T_fr_*(*T_th_*) cooling time, and the estimate of ${\langle t_{f}\rangle }_{T_{\mathrm{fr}}(T_{\mathrm{th}})}$, i.e., of the mean time to the onset of subsequent freezing at the freezer temperature *T_th_*.

**Figure 4:**
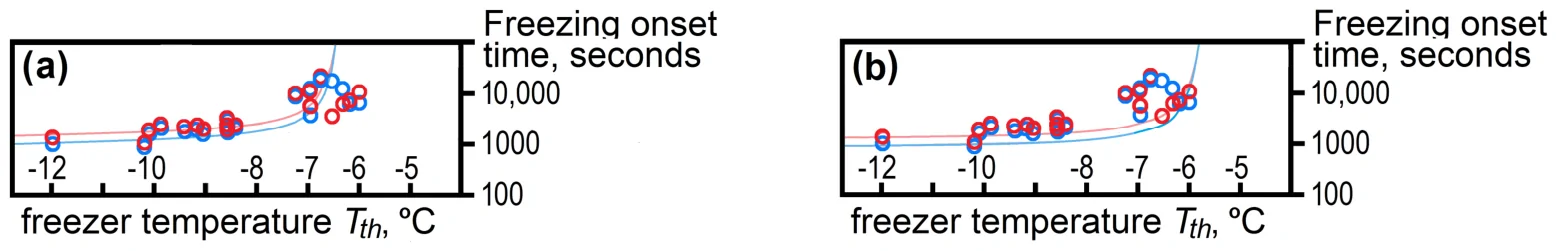
Theoretically expected, from Equations (3) and (11) (pink and light-blue curves for initially hot and cool water, respectively), and experimentally observed (red and blue open circles for initially hot and cool water, respectively; see Table 1a,b) average freezing onset times as a function of the freezer temperature *T_th_*. Each of the 20 experiments used four hot and four cool water samples. The *y*-axis is logarithmic. Calculations were made with *N* = 10^15^, *τ* = 10^−7^ s, *κ* = 0.002 *s*^−1^, and T_*_ = 400° (panel (**a**)); and *N* = 10^15^, *τ* = 10^−7^ s, *κ* = 0.0022 *s*^−1^, and T_*_ = 360° (panel (**b**)).

The total mean time *t_tot_* = $t_{c}\left(T_{\mathrm{th}}\right)+{\langle t_{f}\rangle }_{T_{\mathrm{fr}}(T_{\mathrm{th}})}$ of the cooling and freezing process (i.e., the average freezing onset time) is presented in Figure 4.

The value of *t*_c_ depends on *T_th_* only slightly, whereas the dependence of ${\langle t_{f}\rangle }_{T_{\mathrm{fr}}}$ on it is strong. Therefore, a sharp rise in $t_{c}\left(T_{\mathrm{th}}\right)+{\langle t_{f}\rangle }_{T_{\mathrm{fr}}(T_{\mathrm{th}})}$ at *T_th_* corresponding to ≈−7 °C depends primarily on a sharp rise in the ${\langle t_{f}\rangle }_{T_{\mathrm{fr}}}$ values (given by Equation (11)) at a higher *T_th_*.

Since the value of *T_fr_*(*T_th_*) − *T*_0_ changes in our experiments only by a factor of two (−5 to −10 °C at the most), while the *T_fr_* − *T_th_* may vary by orders of magnitude (from ~0.01° (see lines in Figure 3 at *T_th_* close to −6 °C) to ~5 °C (see lines in Figure 3 at *T_th_* close to −12 °C)), *T_fr_* − *T_th_* has a much stronger impact than *T*_0_ − *T_fr_* on the value of $\frac{[{\left(T_{0}-T_{\mathrm{fr}}\right)/T_{*}]}^{2}}{\left(T_{\mathrm{fr}}-T_{\mathrm{th}}\right)/T_{*}}$.

At $\frac{[{\left(T_{0}-T_{\mathrm{fr}}\right)/T_{*}]}^{2}}{\left(T_{\mathrm{fr}}-T_{\mathrm{th}}\right)/T_{*}}>1$, which is the case when *T_th_* is above ≈−7 °C and *T_fr_* − *T_th_* is thus very small (see Figure 3), $\frac{\tau }{N}\exp\left(\frac{T_{*}}{T_{0}-T_{\mathrm{fr}}}\right)$ exceeds 1κ and grows exponentially with increasing *T_th_* and *T_fr_*. This is the case (A) when the expected freezing onset time ${\langle t_{f}\rangle }_{T_{\mathrm{fr}}(T_{\mathrm{th}})}$ can be exponentially large.

On the contrary, in the case (B) when $\frac{[{\left(T_{0}-T_{\mathrm{fr}}\right)/T_{*}]}^{2}}{\left(T_{\mathrm{fr}}-T_{\mathrm{th}}\right)/T_{*}}<1$, since *T_fr_* − *T_th_* is not as small (which happens when *T_th_* is below −7.2 °C) and τNexpT*T0−Tfr<1κ, then the expected freezing onset time ${\langle t_{f}\rangle }_{T_{\mathrm{fr}}(T_{\mathrm{th}})}$ is relatively small—less than the ~500 s delay in cooling the initially hot water.

Now we can estimate the variance of the expected time of the cooling and freezing process. As known, the expected root mean square deviation (RMSD*_X_*) of experimental *X* values from their mean 〈*X*〉 is $\sqrt{\langle X^{2}\rangle -{\langle X\rangle }^{2}}$, where 〈*X*^2^〉 is the mean square of *X*.

**Figure 5:**
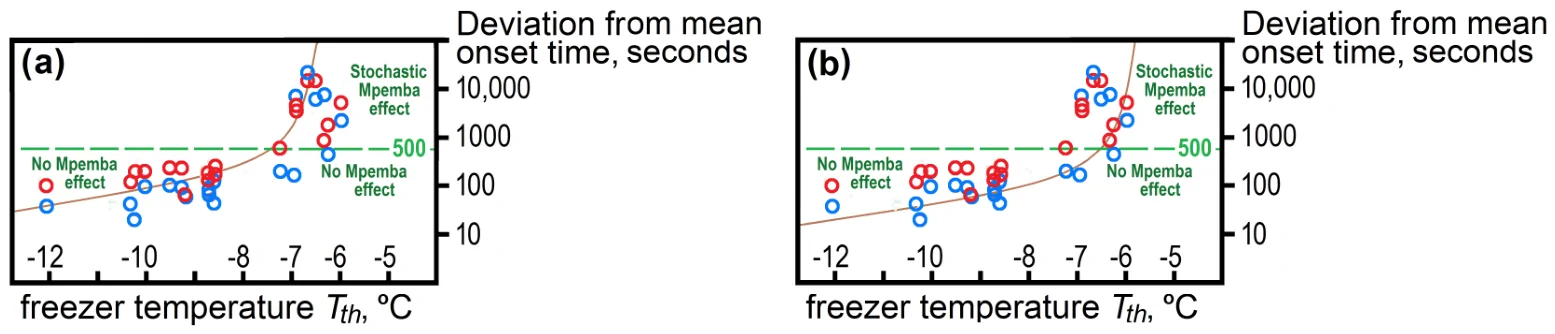
Expected (from Equations (11) and (14), brown curve) and experimentally observed (red and blue open circles for initially hot and cool water, respectively) root mean square deviations (${\mathrm{RMSD}}_{t_{f}}$) of experimental freezing onset times from their average values as a function of the freezer temperature *T_th_*. The green dashed line corresponds to a delay in the cooling of the initially hotter water. The region where the accidental Mpemba effect occurs is above this line. The *y*-axis is logarithmic. Calculations were made with *N* = 10^15^, *τ* = 10^−7^ s, *κ* = 0.002 *s*^−1^, and T_*_ = 400° (panel (**a**)); and *N* = 10^15^, *τ* = 10^−7^ s, *κ* = 0.0022 *s*^−1^, and T_*_ = 360° (panel (**b**)).

The mean square of the freezing onset time ${\left.t_{f}\right|}_{T_{\mathrm{fr}}}$ is

(13) $${\langle t_{f}^{2}\rangle }_{T_{\mathrm{fr}}}=\int_{0}^{\infty }t^{2}\cdot P_{t>0}^{+}\cdot \mathrm{dt}=\int_{0}^{\infty }t^{2}\cdot d[-\exp(-A_{T_{\mathrm{fr}}}t)]=\frac{2}{A_{T_{\mathrm{fr}}}^{2}}\equiv 2{\langle t_{f}\rangle }_{T_{\mathrm{fr}}}^{2},$$

such that the expected variance of the experimental freezing times is

(14) $${\mathrm{RMSD}}_{t_{f}}=\sqrt{{\langle t_{f}^{2}\rangle }_{T_{\mathrm{fr}}}-{\langle t_{f}\rangle }_{T_{\mathrm{fr}}}^{2}}={\langle t_{f}\rangle }_{T_{\mathrm{fr}}}\equiv \frac{\tau }{N}\exp\left(\frac{T_{*}}{T_{0}-T_{\mathrm{fr}}}\right),$$

which is just equal (see Equation (11)) to the mean freezing onset time ${\langle t_{f}\rangle }_{T_{\mathrm{fr}}}$; both drastically grow when *T_th_* (and hence, *T_fr_*) is above −7.2 °C—see Figure 5 (which, like Figure 4, demonstrates satisfactory agreement between the theoretical curves and the experimental points, especially at *κ* = 0.0022 *s*^−1^ and T_*_ = 360°; see panels 4(b) and 5(b)). It should be noted that the fact that the standard deviation of the freezing onset time is equal to the mean freezing onset time is generally typical of the simplest Poisson process (when the expected number of events is zero, just like the number of nucleation events prior to a first-order phase transition).

At the same time, the expected variance of the cooling time *t*_c_(*T_fr_*) is small despite some (small, because ice-nucleating clusters of waters are large [45,49] around 0 °C) stochastic deviations of local temperatures from the mean water temperature *T_fr_* [51]. So, the variance of the cooling time *t*_c_ can be neglected—unlike the variances in the freezing onset times *t*_f_ that can be large and *therefore* significant.

Thus, the resulting estimate of the time for the full *cooling and freezing* process is

(15) $$\left[t_{c}\left(T_{\mathrm{fr}}\right)+{\langle t_{f}\rangle }_{T_{\mathrm{fr}}}\right]\pm {\mathrm{RMSD}}_{t_{f}}\approx \left[\frac{1}{\kappa }\cdot \ln\frac{T_{1}-T_{\mathrm{th}}}{T_{\mathrm{fr}}-T_{\mathrm{th}}}+\frac{\tau }{N}\exp\left(\frac{T_{*}}{T_{0}-T_{\mathrm{fr}}}\right)\right]\pm \frac{\tau }{N}\exp\left(\frac{T_{*}}{T_{0}-T_{\mathrm{fr}}}\right),$$

where the exponential terms dominate when *T_th_* (and hence, *T_fr_*(*T_th_*)) is above −7 °C.

As a result, one expects (and observes in the experiments; see Figure 1 and Table 1a,b) long, highly variable ice-nucleation times when *T_th_* is above −7 °C and short, low-variable ice-nucleation times when *T_th_* is below −8 °C, especially at −10 °C.

## 4. Materials and Methods

In each of our many experiments, we used four “initially cool” and four “initially hot” water samples, all placed in the freezer simultaneously. This simultaneous study of water freezing in many vessels allowed us not only to compare freezing events occurring in vessels with different initial temperatures (which has typically been done in other studies of the Mpemba effect) but also the processes occurring in vessels with the same initial temperatures (which has not been done in other studies of the Mpemba effect).

The experiments were conducted in the basement of a separate building, where all vibration sources were turned off to prevent their impact on ice freezing. For cooling, a Tuvio FCS15HW1 chest freezer (made by the Guangzhou Midea Hualing Refrigerator Co., Ltd., Guangzhou City, Guangdong Province, China) was used; its horizontal lid (see Supplementary Materials S1, Figure S1) minimizes temperature fluctuations during its opening, which contrasts freezers with a vertical door. This freezer allows one to set the temperature from +13 °C to −30 °C with an accuracy of about ±1°. We always used temperatures above −15 °C (this was the temperature at the chest freezer’s bottom). Except for the heat exchanger, the freezer was well-insulated. To further stabilize the temperature, about 30 kg of frozen water in big plastic bottles and about 3.5 kg of frozen water in small plastic bottles were placed in the freezer.

To mitigate temperature fluctuations, we constructed an “inner chamber” within the freezer. This chamber was a foam plastic box (see Supplementary Materials S1, Figures S1 and S2) that housed eleven small plastic bottles, each containing 310 mL of a 15% NaCl solution. Before each experiment, these bottles were pre-cooled to about −12 °C. The gradual thawing of this salt water effectively stabilized the temperature inside the chamber, reducing its fluctuations to less than ±0.5° a day, in contrast to the ±2° fluctuations observed outside the chamber due to the compressor’s cycling.

We initiated each experiment only after the temperature *T_th_* inside the inner chamber, monitored by a thermistor (i.e., a temperature-sensitive resistor connected by a long wire to an external digital thermometer), reached the desired point for this experiment. Within the inner chamber, a lightweight wire stand (see Supplementary Materials S1, Figures S1 and S2) was suspended using 18 thin 100 cm long wires. Nine thermistors, connected to external electronics, were affixed to the ends of these nine wire pairs. Eight of the thermistors were inserted into eight closed 2 mL test vials through holes in their lids. Each vial was thus suspended by a pair of wires, which minimized vibrations, with a thermistor positioned at the end of the wire pair. Water occupied 80% ± 1% of the vial volume, and the thermistor measured the temperature in the upper half of the liquid.

The assignment of the same physical vial to the hot/cool groups and to different positions was randomized between runs.

The vials and their contents were weighed with a precision of 10 mg. Four vials containing the initially hot water (+70 °C) and four containing the initially cool water (+20 °C) were arranged on the wire stand in an alternating pattern, separated by 4 cm. All vials were placed into the freezer simultaneously. The ninth thermistor was placed in the air between the salt water bottles and test vials to monitor the ambient temperature.

For temperature measurements, we used nine MF59 100kΩ thermistors (made by the Hefei XIXI Electronics Co., Ltd (XIXITRONICS), Hefei, Anhui, China, 230088), each with a 1 m long wire. These thermistors were pre-calibrated using a digital thermometer with a resolution of 0.01° (LT-300, made by the Termexlab in Tomsk, Russia). Eight of these thermistors were used to monitor the water temperature within the test vials, while the ninth measured the air temperature inside the inner chamber containing the small bottles with salt semi-frozen water.

Additionally, we used pre-calibrated digital thermometers (of 0.1° resolution) with sensors at the ends of the 1 m long wires to monitor the temperature inside the inner chamber, the temperature of ambient air, and the temperature of the electrical heater used for pre-heating the “initially hot” water samples.

These two digital thermometers, DT1 and DT2 (made by the Shanghai QualityWell. No. 647 Taibo Road, Fangtai, Jiading District, Shanghai, China, 201814), were used to monitor the temperature throughout the experiment, especially during the process of water freezing (when the temperature of the water/ice mixture was close to 0 °C), and after the freezing has been completed (when the temperature of the formed ice approached the temperature inside the inner chamber). These data were used for the final calibration of all thermistors.

We used a custom-built data acquisition module that included a computer running specialized software, which sent 5V pulses sequentially to each of the nine thermistors via the LPT1 parallel port and a custom adapter (see Supplementary Materials S1, Figure S3). Digital voltage readings, representing the temperature, were collected from the Radio Shack 22-168A digital multimeter (Custom manufactured in Korea for the Radio Shack division of the Tandy Corporation, Fort Worth, TX, 76102, USA, Date 1993) connected to the RS232 serial port of computer HP Compaq dc5100 MT with Window XP, SP3, and Visual Basic 6.0. This multimeter measured the voltage across a single 100 kΩ resistor (shared by all thermistors and connected to ground), the current through which the temperature of the thermistor depended on.

Data regarding voltage and corresponding timestamps were recorded at 43 s intervals (see Supplementary Materials S1, Figure S4); thus, the uncertainty in time measurements was ±22 s. The collected voltage data were subsequently converted into temperature data (see Supplementary Materials S1, Figure S5). The maximum expected error in temperature measurements (including the thermistor calibration uncertainty) was ±0.5°. This level of precision is adequate for the objectives of this study, as the temperature increase associated with the onset of freezing exceeds 5° and the point of our interest was the freezing onset time.

All experiments were conducted using distilled water from the Institute of Protein Research, Russian Academy of Sciences. Before each run, four closed vials were equilibrated at the ambient basement temperature (+15 ÷ +20 °C), while four others were pre-heated to the temperature of ≈+70 °C using small custom-built electric ovens.

The interiors of the test vials and thermistors in the vials were lubricated with “Sylgard 184 silicone elastomer” (without the “Curing Agent”; made by Dow, 3901 S Saginaw Rd, Midland, MI 48640, USA) to adsorb potential foreign ice nucleators from the water and to smooth out any micro-roughness on the vial walls.

Upon placing the wire stand with vials into the freezer, a small temporary temperature increase was observed in the upper part of the chamber. However, the air temperature, as monitored by a digital thermometer and the intra-chamber thermistor, returned to the setpoint within 10 min—well before freezing onset.

## 5. Conclusions

We see that the Mpemba effect in water freezing (at least under the given conditions of closed vessels that prevent evaporation, suppress convection, and exclude the entry of alien crystallization centers in water) arises *not* from any special properties of pure water (as has been hypothesized in many papers on this topic) or of some in-water “motes” [39] but from the general uncertainty of the time to the onset of the first-order phase transition—an uncertainty that becomes especially significant at slightly sub-zero water temperatures.

This fundamental uncertainty (which does not allow one to precisely define the onset time) is well known [51] in the physics of first-order phase transitions, yet oddly enough, it has never been, to our best knowledge, considered in the context of the Mpemba effect in the freezing of liquids, even though it is precisely here that this uncertainty manifests itself perhaps most clearly.

It is this fundamental uncertainty in the freezing onset time (which has been mentioned but not investigated in [2,39,52,53]) that produces a kind of randomness in the occurrence of the Mpemba effect, and its poor reproducibility mentioned in many works, which, for many years, has “continued to make the Mpemba effect as puzzling as ever” [53] and generated skepticism about its very existence [28].

The large variability in the onset times is due to the specific (Poisson) form of the time-dependent probability of freezing transitions, $P_{t>0}^{+}\sim \exp(-A_{T_{\mathrm{fr}}}t)$. $P_{t>0}^{+}$ has its maximum at the first moment and then declines. This leads to substantial uncertainty in the onset timing and, at sufficiently high *T_th_*, results in the overlap of freezing times for samples with different initial temperatures, thus providing the stochastic Mpemba effect.

The observed stochasticity of the Mpemba effect in freezing of pure water does not exclude other factors [54] that may contribute to the freezing times, e.g., insufficiently clean liquid or vessel walls (we minimized these effects; see Materials and Methods), or ice-nucleating “motes” that can fall in open vials positioned in a freezer. Therefore, the Mpemba effect is not caused by the liquid itself but may result from some foreign ice nucleators penetrating into the initially hot water [15].

It is important to emphasize that the observed accidental origin of the Mpemba effect in pure water is limited to phenomena such as freezing, which involves first-order phase transitions.

The initiation of freezing is, in essence, a catastrophe [55], or “a single-event phenomenon”: the emergence of a single ice nucleus can trigger the freezing of the entire liquid in the test tube. Therefore, the significant uncertainty in the timing of a stochastic ice nucleation can occasionally demonstrate the Mpemba effect in *some* experiments. This occurs due to the overlap of broad ranges of ice initiation times for initially hot and initially cool samples. This overlap (see Figure 2c,d) can mask the delay in cooling of the hotter water sample and leading to the wrong perception that “hot water [deterministically, always] freezes faster than cool water”, or, according to a modern, more general formulation of the Mpemba paradox, that “the state that is initially farthest from its equilibrium state attains the latter at the earliest time” [13].

We do not want to create the impression that our finding of the stochasticity of the Mpemba effect in freezing refutes the very existence of deterministic Mpemba effects altogether. Colloids [8,9,11], simple mechanical systems [40,41], and specifically various oscillators (see Supplementary Materials S3 and [56,57]) exhibit a deterministic Mpemba effect, where “the state that is initially farthest from its equilibrium state (always) attains the latter at the earlier [56] (or at least the same [57]) time”, and where the origin and driving force of this effect (inertia—“kinetic memory”—and a sharper than quadratic increase in potential upon deviation from equilibrium) are clear (as we show in Supplementary Materials S3).

In essence, all discussions regarding the Mpemba effect in various systems boil down to attempts to identify a hidden, stable ordering parameter (or parameters)—e.g., currents—in addition to such familiar quantities as temperature, volume, and pressure, much like what has been done for the Kovacs effect [31,58,59,60]. This effect was observed when the polymer glass—having been brought into a non-equilibrium state, where its macroscopic thermodynamic parameters (temperature, density) nevertheless coincide with their equilibrium values—continues to evolve and exhibits a temporal “hump” before transitioning to a fully equilibrium state.

Discussions regarding the Mpemba effect now apply particularly to anomalous relaxation behaviors of open and isolated nonequilibrium quantum systems [61,62,63,64,65,66,67], whose “resource” is an asymmetrical thermal or athermal deviation from symmetry or thermal equilibrium. Most of these studies employ a quantum information-theoretic approach based on the resource theory of asymmetry and quantum relative entropy, and bear little relation to first-order thermodynamic phase transitions of the freezing type.

Ironically, the classic Mpemba effect, a phenomenon first found just in the *freezing of water* sixty years ago, proved to be only stochastic in this system, whereas other systems—namely those *lacking a first-order phase transition* (colloid, quantum, etc.)—can exhibit a fully deterministic Mpemba effect.

## Figures and Tables

**Table 1:** (**a**) Data on cooling and freezing ^1^ at thermostat temperatures *T_th_* of −7.2 °C and higher. (**b**) Data on cooling and freezing ^1^ at thermostat temperatures *T_th_* below −8 °C.

(**a**)								
***T_th_*** **, °C**	**<*t*_hot_** − ***t*_cool_> Delay** **at 0° C**	**Initially**	**Time of the 1st Freezing Onset ^2^**	**Time of the 2nd Freezing Onset ^2^**	**Time of the 3rd Freezing Onset ^2^**	**Time of the 4th Freezing Onset ^2^**	**Hot** **Before** **Cool**	**Cool** **Before** **Hot**
−6.3 ±0.1	456 ± 98	cool	3453	4686	14,673	21,120	0; 1; 4; 4; ^3^ ∑: 9 (56%)	1; 2; 2; 2; ^4^ ∑: 7 (44%)
		hot	4562	5980	6412	6658		
−6.9 ±0.1	353 ± 43	cool	2959	3243	17,139	17,262	0; 0; 4; 4; ^3^ ∑: 8 (50%)	2; 2; 2; 2; ^4^ ∑: 8 (50%)
		hot	3576	10,604	10,974	11,985		
−6.9 ±0.1	356 ± 123	cool	3453	3699	3835	3946	1; 2; 2; 2; ^3^ ∑: 7 (56%)	0; 1; 4; 4; ^4^ ∑: 9 (44%)
		hot	3206	3576	4229	12,330		
−6.0 ±0.1	400 ± 43	cool	4720	4914	5920	9671	0; 0; 1; 3; ^3^ ∑: 4 (25%)	2; 3; 3; 4; ^4^ ∑: 12 (75%)
		hot	5766	6577	6693	18,460		
−6.2 ±0.1	520 ± 33	cool	4345	5119	5378	5463	0; 0; 1; 1; ^3^ ∑: 2 (12%)	2; 4; 4; 4; ^4^ ∑: 14 (88%)
		hot	5334	6409	7012	10,046		
−6.5 ±0.2	364 ± 103	cool	6240	14,459	17,170	22,676	2; 3; 3; 3; ^3^ ∑: 11 (69%)	0; 0; 1; 4; ^4^ ∑: 5 (31%)
		hot	5725	6155	8522	40,535		
−6.7 ±0.2	309 ± 178	cool	4210	6532	8261	53,906	0; 1; 2; 4; ^3^ ∑: 7 (44%)	1; 2; 3; 3; ^4^ ∑: 9 (56%)
		hot	4913	6661	9759	50,096		
−7.2 ±0.2	399 ± 76	cool	7827	8128	8300	8343	0; 0; 1; 1; ^3^ ∑: 2 (12%)	2; 4; 4; 4; ^4^ ∑: 14 (88%)
		hot	8171	8472	8601	9634		
							Mean: 39%	Mean: 61% ^5^
(**b**)								
***T_th_*** **, °C**	**<*t*_hot_** − ***t*_cool_> Delay** **at 0° C**	**Initially**	**Time of the 1st Freezing Onset ^2^**	**Time of the 2nd Freezing Onset ^2^**	**Time of the 3rd Freezing Onset ^2^**	**Time of the 4th Freezing Onset ^2^**	**Hot** **Before** **Cool**	**Cool** **Before** **Hot**
−8.6 ±0.1	532 ± 58	cool	1922	2010	2064	2242	0; 0; 0; 0; ^3^ ∑: 0 (0%)	4; 4; 4; 4; ^4^ ∑: 16 (100%)
		hot	2411	2463	2644	2778		
−8.7 ±0.1	398 ± 28	cool	1716	1828	1840	1942	0; 0; 0; 0; ^3^ ∑: 0 (0%)	4; 4; 4; 4; ^4^ ∑: 16 (100%)
		hot	2221	2332	2410	2901		
−12.0 ±0.1	432 ± 24	cool	1276	1341	1420	1444	0; 0; 0; 0; ^3^ ∑: 0 (0%)	4; 4; 4; 4; ^4^ ∑: 16 (100%)
		hot	1516	1618	1685	1784		
−8.7 ±0.3	400 ± 33	cool	1397	1397	1441	1441	0; 0; 0; 0; ^3^ ∑: 0 (0%)	4; 4; 4; 4; ^4^ ∑: 16 (100%)
		hot	1527	1613	1914	1957		
−8.7 ±0.2	520 ± 73	cool	2135	2177	2221	2264	0; 0; 0; 0; ^3^ ∑: 0 (0%)	4; 4; 4; 4; ^4^ ∑: 16 (100%)
		hot	2393	2522	2737	3168		
−9.2 ±0.1	510 ± 29	cool	1433	1476	1543	1606	0; 0; 0; 0; ^3^ ∑: 0 (0%)	4; 4; 4; 4; ^4^ ∑: 16 (100%)
		hot	1735	1822	1864	1950s		
−9.2 ±0.3	314 ± 53	cool	1849	1852	2011	2107	0; 0; 1; 2; ^3^ ∑: 3 (19%)	2; 3; 4; 4; ^4^ ∑: 13 (81%)
		hot	1853	2017	2407	2493		
−9.4 ±0.1	482 ± 17	cool	1624	1667	1797	1926	0; 0; 0; 1; ^3^ ∑: 1 (6%)	3; 4; 4; 4; ^4^ ∑: 15 (94%)
		hot	1883	1969	2443	2486		
−9.9 ±0.1	420 ± 23	cool	1892	1935	2063	2150	0; 0; 1; 1; ^3^ ∑: 2 (12%)	2; 4; 4; 4; ^4^ ∑: 14 (88%)
		hot	1978	2193	2450	2536		
−10.2 ±0.1	455 ± 39	cool	1453	1495	1495	1581	0; 0; 0; 0; ^3^ ∑: 0 (0%)	4; 4; 4; 4; ^4^ ∑: 16 (100%)
		hot	1624	1667	1753	1968		
−10.2 ±0.2	564 ± 53	cool	1666	1709	1709	1709	0; 0; 0; 0; ^3^ ∑: 0 (0%)	4; 4; 4; 4; ^4^ ∑: 16 (100%)
		hot	1924	1924	2059	2183		
							Mean: 3%	Mean: 97% ^5^

In (**a**): ^1^ Numbers in lines 1–2, 3–4, and 5–6 of the Table correspond to Figure 1a. ^2^ Temperatures for the freezing onset (ice nucleation) in individual water samples are ≈−6.0 ± 0.6 °C. ^3^ The four integers in this line indicate the number of “hot” samples that begin to freeze before the “cool” samples, corresponding to the first, second, third, and fourth freezing onsets. ^4^ The four integers in this line indicate the number of “cool” samples that begin to freeze before the “hot” samples corresponding to the first, second, third, and fourth freezing onsets. ^5^ A comparison of the order in which freezing onsets occur for “initially cold” and “initially hot” water samples is provided in Supplementary Materials S2. In (**b**): ^1^ Numbers in lines 1–2, 3–4, and 5–6 of the Table correspond to Figure 1b. ^2^ Temperatures for the freezing onsets (ice nucleation) in individual water samples are ≈−6.1 ± 0.5 °C. ^3^ The four integers in this line indicate the number of “hot” samples that begin to freeze before the “cool” samples corresponding to the first, second, third, and fourth freezing onsets. ^4^ The four integers in this line indicate the number of “cool” samples that begin to freeze before the “hot” samples corresponding to the first, second, third, and fourth freezing onsets. ^5^ A comparison of the order in which freezing onsets occur for “initially cold” and “initially hot” water samples is provided in Supplementary Materials S2.

## Data Availability

The original contributions presented in this study are included in the article. Further inquiries can be directed to A.V.F. (theory) or A.A.K. (experimentation).

## Supplementary Materials

The following supporting information can be downloaded at https://www.mdpi.com/article/10.3390/molecules31213954/s1, Figure S1. Experimental setup for water freezing experiments; Figure S2. Wire stand with closed vials containing water samples and thermistors; Figure S3. Scheme of the custom-built adapter; Figure S4. Example of the program interface window used for data acquisition; Figure S5. Example of the program output; Figure S6. Illustration of the competition between the kinetics of two Poisson processes, where the second one begins with a time lag Δ*t* relative to the first; Figure S7. Potential energy for a system where “a particle located between two other particles” exhibits a Mpemba-like effect in the absence of a phase transition.
