# Supplementary material for: Accidental Origin of the Mpemba Effect in Freezing: Reversals in the Freezing Order Are Stochastic—Experimentation and Theory
Source: Molecules. 2026 Nov 2;31(21):3954. doi: 10.3390/molecules31213954 (PMC13765033; doi:10.3390/molecules31213954)
Supplement: Supplementary file 1 [file molecules-31-03954-s001.zip › molecules-4570034-supplementary.pdf]

# Supplementary Materials

## 1. Stochastic origin of the Mpemba effect in water freezing: Experimental setup

Based on numerous experiments comparing the freezing onset time of initially cool and initially hot water, we developed the following requirements for the experimental protocol:

1) The temperature of at least 4 vials with initially hot water and 4 vials with initially cool water, and the temperature of the surrounding "freezing" air must be measured simultaneously. All temperature and corresponding time data are logged into a computer file.

2) The temperature of the air “freezing” the water in the vials should not fluctuate by more than ±0.5 ºC over many hours of the experiment.

3) The vibration of the vials containing the water samples must be minimized, as any disturbance can accelerate the crystallization process.

4) To prevent the cold air in the freezer from being replaced by warmer room air when placing the samples inside the freezer, we utilized a chest freezer with a horizontal lid and an additional inner chamber housing these vials (see Figs. S1, S2).

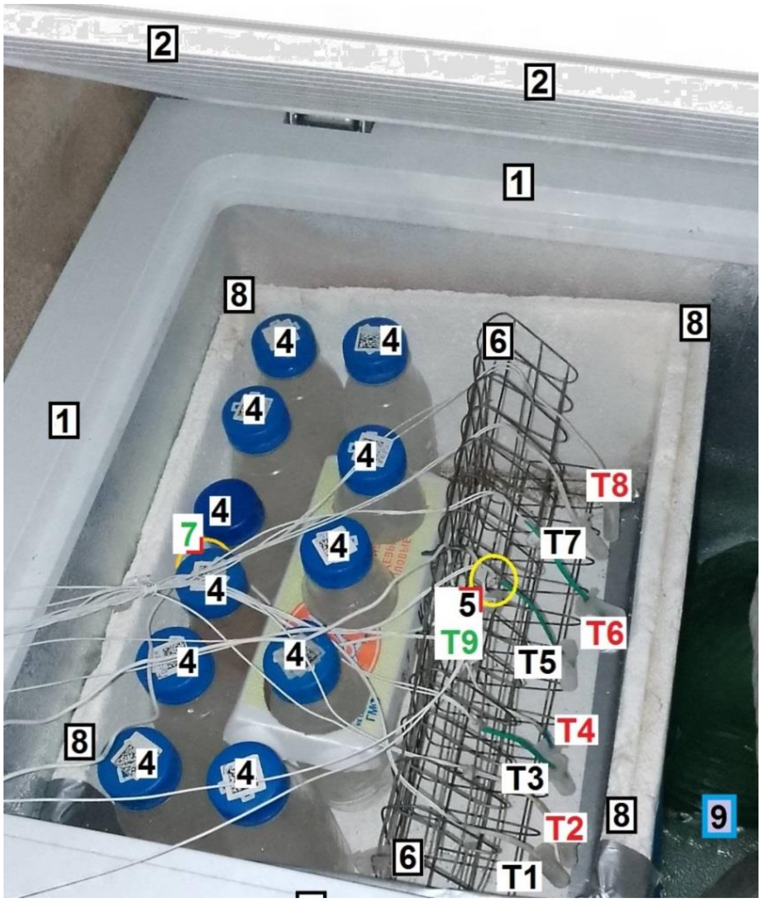


**Figure S1.** Experimental setup. A FCS15HV1 chest freezer (1) with its horizontal lid (2) (opened), and external thermal insulation (3) applied; 11 small bottles (4) each containing 310 mL of a semi-frozen 15% NaCl solution; a thermistor (5) of the external digital thermometer DT1, combined with thermistor T9, is positioned in the air on the wire stand (6) and situated between the small salt water bottles (4), and the vials with thermistors T1-T8 containing initially hot (T1, T3, T5, T7) and initially cool (T2, T4, T6, T8) water; a thermistor (7) of the external digital thermometer DT2 placed in the air behind the small bottles (4) with semi-frozen salt water. These small bottles, the wire stand, the vials, and the thermistors are housed within the foam box (8), which serves as an "inner chamber." The wire stand (6) serves as a support for the thin thermistor wires. To reduce the influence of the compressor vibrations, the thermistors (placed in water inside closed vials T1-T8) are at a distance of 8 cm from the wire stand. Large plastic bottles with frozen water (9) are also present in the freezer to increase the time between freezer compressor starts.

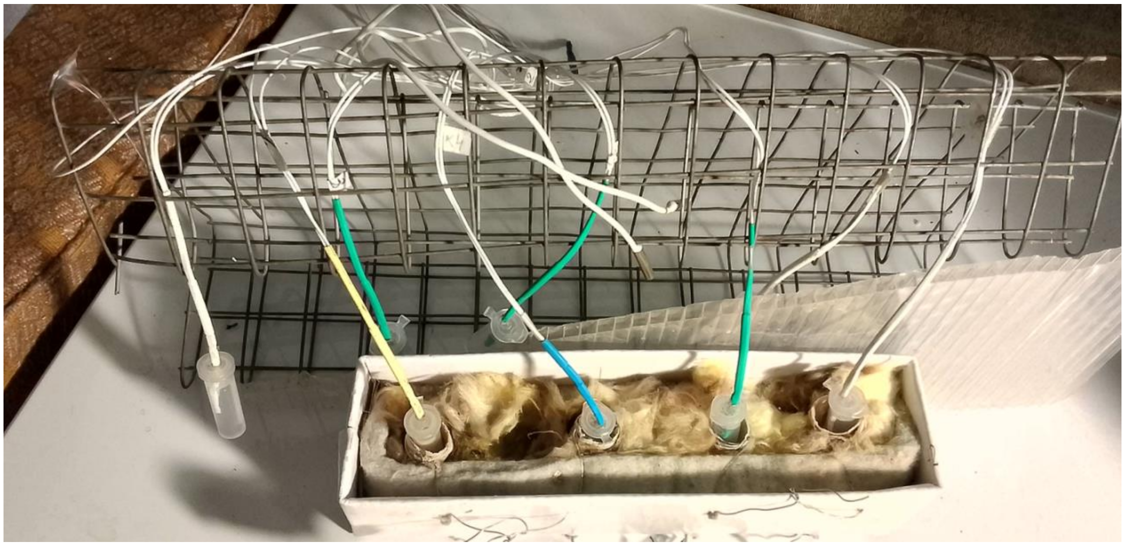

**Figure S2.** A wire stand with vials containing water samples. 4 vials contain water, the initial temperature of which corresponds to the air temperature in the room. The other 4 water-filled vials (which are now hanging in the air) are to be heated in electric heaters before being placed in the foam box inside the chest freezer.

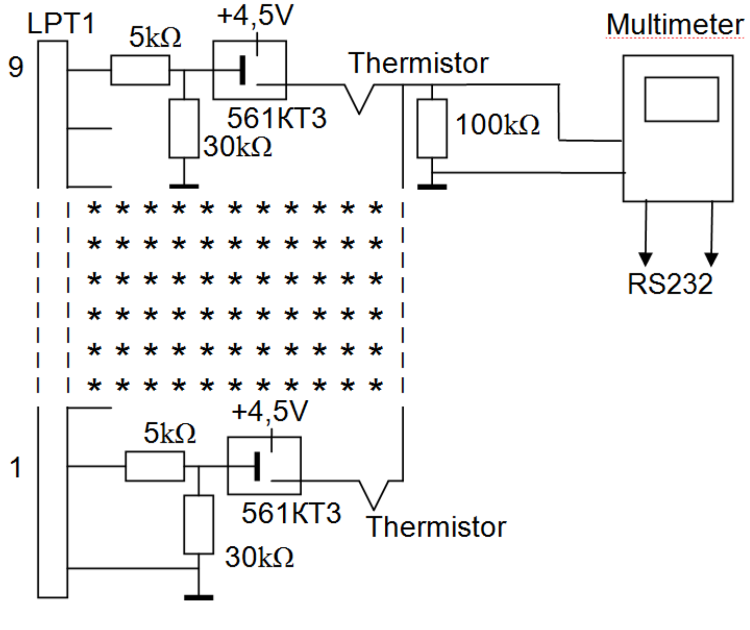


**Figure S3.** A scheme of the custom-built adapter. This adapter is designed to generate and apply electrical impulses sequentially to 9 thermistors (T1-T8, T9; see Fig. S1). The impulses are transmitted from a computer's LPT1 parallel port to the adapter. For clarity, only 2 of the 9 thermistor circuits are shown. Digital voltage readings were taken from the Radio Shack 22-168A digital multimeter connected to the RS232 serial port of the same computer. This multimeter measured the voltage across a single 100 kΩ resistor, which is common to all thermistors and connected to ground. The current flowing through the resistor is directly dependent on the thermistor's temperature.

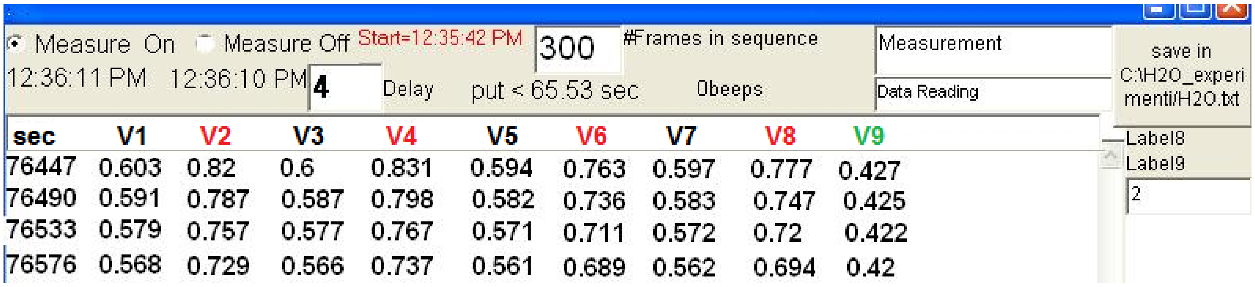


| sec | V1 | V2 | V3 | V4 | V5 | V6 | V7 | V8 | V9 |
|---|---|---|---|---|---|---|---|---|---|
| 76447 | 0.603 | 0.82 | 0.6 | 0.831 | 0.594 | 0.763 | 0.597 | 0.777 | 0.427 |
| 76490 | 0.591 | 0.787 | 0.587 | 0.798 | 0.582 | 0.736 | 0.583 | 0.747 | 0.425 |
| 76533 | 0.579 | 0.757 | 0.577 | 0.767 | 0.571 | 0.711 | 0.572 | 0.72 | 0.422 |
| 76576 | 0.568 | 0.729 | 0.566 | 0.737 | 0.561 | 0.689 | 0.562 | 0.694 | 0.42 |

**Figure S4**. An example of the program interface window used for data acquisition. The first column displays the time in seconds, measured from midnight. The subsequent 9 columns show the thermistor T1-T9 voltages, as measured by Radio Shack 22-168A digital multimeter. Red symbols refer to initially hot waters, black symbols refer to initially cool waters.

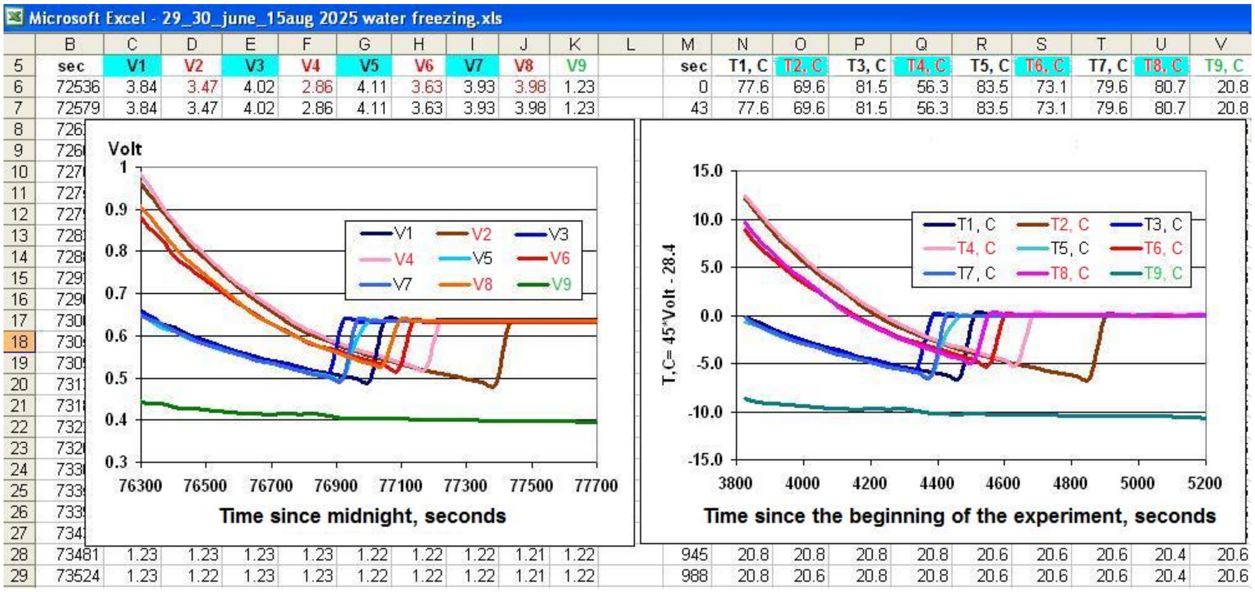


**Figure S5.** An example of the output. In the background: An example of window with the data of our program (first 10 columns, see Fig. S4), and, in the next 10 columns: the calculated values of time after the start of the experiment (first column) and the thermistor T1-T9 temperatures (next 9 columns). In the foreground: graphs of the time dependence of the measured voltages (left) and the corresponding thermistor temperatures (right). Red symbols refer to initially hot waters, black symbols refer to initially cool waters. The formula T(ºC) = 45×Volt - 28.4 is used for the temperature range from -12 ºC to +12 ºC. For the temperature range from +12 ºC to +80 ºC, the formula T(ºC) = 21.8×Volt - 6.0 is used.

## 2. "Cool-before-hot" *vs.* "hot-before-cool" in freezing

Since "initially hot" water reaches the freezing temperature $T_{fr}$ later than "initially cool" water (with a delay $\Delta t$ of approximately 500 seconds), it makes sense to consider the competition between the kinetics of two independent, identical Poisson processes that lead to the onsets of freezing for "initially cool" and "initially hot" water, where the second process begins with a time lag $\Delta t$ relative to the first one (Figure S6).

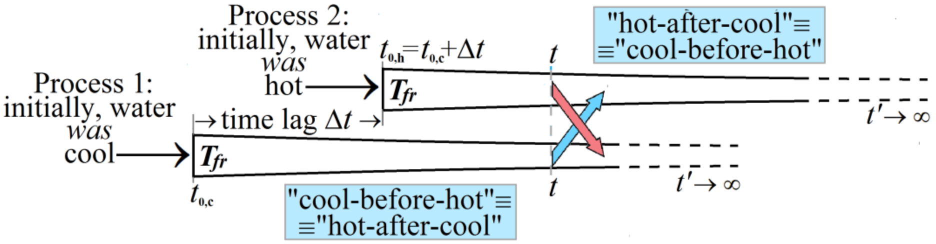


**Figure S6**. An illustration of the competition between the kinetics of two Poisson processes of ice nucleation at a fixed sub-zero water freezing temperature $T_{fr}$, where the second process—due to the longer cooling time required for hotter water—begins with a time delay $\Delta t$ relative to the first one. $t_{0,c}$ is the time of achieving the temperature $T_{fr}$ by the "initially cool" water sample, $t_{0,h}$ is the time of achieving the temperature $T_{fr}$ by the "initially hot" water sample ($t_{0,c} < t_{0,h}$).

According to Eqs. (10, 11) of the main text, the momentary probability of ice nucleation at time $t > t_0$ ($t_0$ being the time point at which a given water sample reaches the temperature $T_{fr}$) is

$$P^+_{t-t_0} = A_{T_{fr}}\, exp\left[-A_{T_{fr}}(t - t_0)\right],$$

(note: $P^+_{t-t_0}= 0$ at $t < t_0$, and $\int_{t_0}^{\infty} P^+_{t-t_0} dt = 1$). $A_{T_{fr}} = \frac{N}{\tau} exp\left(\frac{-T_*}{T_0 - T_{fr}}\right)$ is the rate constant for ice nucleation at temperature $T_{fr}$; here $N$~$10^{15}$ is the number of $H_2O$ molecules located near the walls of the ~1 mL test tube (since, as noted in the main text, the walls of the test tube facilitate the formation of ice nucleus, whereas the bulk of the water does not); $\tau = 10^{-7}$ sec is the typical time for one $H_2O$ molecule to attach to ice at temperatures of $-1$ to $-20$ °C; $T_0$≡273 K, i.e. 0 °C; $T_* \approx 400^{\mathrm{o}}$ is determined by the free energy of the ice/water interface (see main text); and the water freezing temperature $T_{fr}$, typically corresponds to $-6 \div -7.4$ °C (see the main text: $T_{fr}$ corresponding to $-6$ °C arises at the freezer temperature of $\approx -6$ °C, and $T_{fr}$ corresponding to $-7.4$ °C arises at the freezer temperature of $\approx -12$ °C, see Figure 3). As a result, $A_{T_{fr}}$ varies from approximately $10^{-7}$ at $\approx -6$ °C to approximately 0.03 $sec^{-1}$ at $\approx -7.4$ °C.

It is worth analysing the proportion of "initially cool" and "initially hot" water samples that freeze first at various freezing temperatures $T_{fr}$.

The fraction $F_{c-b-h}$ of "initially cool" water samples that freeze before "initially hot" water samples is

$$F_{c-b-h} = \int_{t_{0,c}}^{t_{0,h}} dt \cdot \left(P^+_{t-t_{0,c}} \cdot 1\right) + \int_{t_{0,h}}^{\infty} dt \cdot \left(P^+_{t-t_{0,c}} \cdot \int_t^{\infty} dt' \cdot P^+_{t'-t_{0,h}}\right) =$$

$$\int_{t_{0,c}}^{t_{0,h}} dt \cdot A_{T_{fr}}\, exp\left[-A_{T_{fr}}(t - t_{0,c})\right] + \int_{t_{0,h}}^{\infty} dt \cdot \left\{A_{T_{fr}}\, exp\left[-A_{T_{fr}}(t - t_{0,c})\right] \cdot \int_t^{\infty} dt' \cdot A_{T_{fr}}\, exp\left[-A_{T_{fr}}(t' - t_{0,h})\right]\right\}.$$

Elementary calculations ($\int_{t_{0,c}}^{t_{0,h}} dt \cdot A_{T_{fr}}\, exp\left[-A_{T_{fr}}(t - t_{0,c})\right] = 1 - \boldsymbol{exp}\left[-\boldsymbol{A_{T_{fr}}\Delta t}\right]$; $\int_t^{\infty} dt' \cdot A_{T_{fr}}\, exp\left[-A_{T_{fr}}(t' - t_{0,h})\right] = exp\left[-A_{T_{fr}}(t - t_{0,h})\right]$; $\int_{t_{0,h}}^{\infty} dt \cdot \left\{A_{T_{fr}}\, exp\left[-A_{T_{fr}}(t - t_{0,c})\right] \cdot exp\left[-A_{T_{fr}}(t - t_{0,h})\right]\right\} = \frac{1}{2} exp\left[-A_{T_{fr}}\Delta t\right]$ make it possible to obtain:

$$F_{c-b-h} = 1 - \frac{1}{2} exp\left[-A_{T_{fr}}\Delta t\right]$$

In a similar way, one can determine the fraction of "initially hot" water samples that freeze before "initially cool" water samples:

$$F_{h-b-c} = \int_{t_{0,\mathrm{h}}}^{\infty} dt \cdot \left( P^{+}_{t-t_{0,\mathrm{h}}} \cdot \int_{t}^{\infty} dt' \cdot P^{+}_{t'-t_{0,\mathrm{c}}} \right) = \frac{1}{2} exp\left[-A_{T_{fr}} \Delta t\right];$$

and, naturally, $F_{c-b-h} + F_{h-b-c} = 1$.

For the set of "higher" freezer temperatures $T_{th}$ ($-6.0$ to $-7.2$ °C, corresponding to the water freezing temperatures $T_{fr}$ of $-6.0$ to $-7.0$ °C, see Table 1a and Figure 3), we have the following experimental data: "Hot-before-cool": 39%; "Cool-before-hot": 61%. From this, the above Equation $F_{h-b-c} = \frac{1}{2} exp\left[-A_{T_{fr}} \Delta t\right]$, and the fact that $\Delta t \approx 500$ sec, we obtain $\frac{1}{2} exp\left[-A_{T_{fr}} \Delta t\right] = 0.39$. Consequently, $A_{T_{fr}} = -\ln(0.39 \cdot 2)/(500 \text{ sec}) \approx 0.0005 \text{ sec}^{-1}$. Given that $A_{T_{fr}} = \frac{N}{\tau} exp\left(\frac{-T_*}{T_0 - T_{fr}}\right)$, and using the values $N \sim 10^{15}$, $\tau \sim 10^{-7}$ sec, $T_* \approx 400^{\circ}$, we obtain: $T_0 - T_{fr} = -T_*/ln\left(\frac{A_{T_{fr}} \tau}{N}\right) \approx -6.8$ °C. This value falls within the first investigated range of "higher" water freezing temperatures $T_{fr}$ ($-6.0$ to $-7.0$ °C).

For the set of "lower" freezer temperatures $T_{th}$ ($-8.6$ to $-12.0$ °C, corresponding to the water freezing temperatures $T_{fr}$ of $-7.2$ to $-7.4$ °C, see Table 1b and Figure 3), we have the following experimental data: "Hot-before-cool": 3%; "Cool-before-hot": 97%. From this, the above Equation $F_{h-b-c} = \frac{1}{2} exp\left[-A_{T_{fr}} \Delta t\right]$, and the fact that $\Delta t \approx 500$ sec, we obtain $\frac{1}{2} exp\left[-A_{T_{fr}} \Delta t\right] = 0.03$. Consequently, $A_{T_{fr}} = -\ln(0.03 \cdot 2)/(500 \text{ sec}) \approx 0.006 \text{ sec}^{-1}$. Given that $A_{T_{fr}} = \frac{N}{\tau} exp\left(\frac{-T_*}{T_0 - T_{fr}}\right)$, and using the values $N \sim 10^{15}$, $\tau \sim 10^{-7}$ sec, $T_* \approx 400^{\circ}$, we obtain: $T_0 - T_{fr} = -T_*/ln\left(\frac{A_{T_{fr}} \tau}{N}\right) \approx -7.2$ °C. This value falls within the investigated range of "lower" water freezing temperatures $T_{fr}$ ($-7.2$ to $-7.4$ °C).

We are grateful to the anonymous reviewer for drawing our attention to this issue.

# 3. The Mpemba pendulum: When a longer path requires less time

A deterministic "Mpemba-like" behavior is generally inherent to various simple physical systems. Among them, we should allude to well-known harmonic pendulums, and especially mention nonlinear oscillators whose dynamic behavior is described by the Duffing equation [57].

As for harmonic pendulums, whose potential energy $U(x)$ is proportional to $x^2$, the squared deviation from the equilibrium, they are famous for the fact that their *initial state, no matter how far it is from equilibrium, transitions to the latter in the same amount of time* [58]. This is already, so to speak, a "zero-order Mpemba effect." And since it is also well known that the time of free fall (when the potential energy $U(x)$ is proportional to the height $x$) *increases* [58] with the initial height $X_0$, it can be assumed that when the potential energy $U(x)$ is proportional to $|x|^{>2}$, the motion starting from a position further from the equilibrium point will reach it earlier; that is, such a "pendulum" (with $U(x) \propto |x|^{>2}$) should exhibit the Mpemba effect.

Indeed, since – for a friction-less motion – the law of conservation of energy reads that $\frac{m}{2}\left(\frac{dx}{dt}\right)^2 + U(x) = U(X_0)$, where $m$ is the "pendulum" mass, $\frac{dx}{dt}$ is its velocity, $U(x)$ is its potential energy, and $X_0 = x|_{t=0}$ is its starting position, we have $\frac{dx}{dt} = -\sqrt{2/m}\cdot\sqrt{\Delta U(X_0) - \Delta U(x)}$, and thus the time of a descent from $X_0$, where $\left.\frac{dx}{dt}\right|_{t=0} = 0$, to the equilibrium (*) point $x|_{t=t^*} = 0$ is

$$t^* \equiv \int_0^{t^*} dt = \frac{-1}{\sqrt{2/m}} \int_{X_0}^{X_{t^*}} \frac{dx}{\sqrt{U(X_0)-U(x)}} = \frac{X_0}{\sqrt{(2/m)\cdot U(X_0)}} \int_{X_{t^*}}^{X_0} \frac{\frac{dx}{X_0}}{\sqrt{1-U\left(X_0\cdot\left(\frac{x}{X_0}\right)\right)/U(X_0)}}.$$

With $U(x) = A|x|^{2+a}$ (where $a$>0), and replacing $\frac{x}{X_0}$ with $z$, we have

$$t^* = \frac{{X_0}^{-a/2}}{\sqrt{2/m}} \int_0^1 \frac{\cdot dz}{\sqrt{1-z^{2+a}}}.$$

Now we see that the time $t^*$ of coming to the equilibrium point *<u>decreases with increasing</u>* initial displacement $X_0$ from this point. This means a " nonlinear pendulum" [57] with $U(x) \propto |x|^{>2}$ exhibits a *<u>deterministic</u>* Mpemba-like effect.

Among such oscillators, a special interest for molecular science is presented by a nonlinear oscillator consisting of a particle located between two other particles (Figure S7).

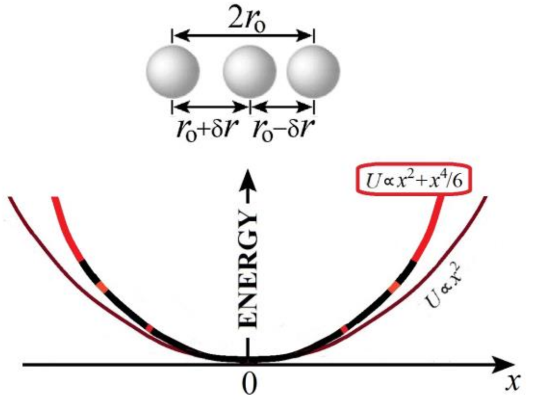


Figure S7. The increase in potential energy of a particle located between two other particles when it deviates from the equilibrium point between them. The thick black/red curve shows the potential energy $U \propto x^2 + x^4/6$ of this system of particles compared to the potential energy $U \propto x^2$ of a harmonic pendulum (shown by the thin brown curve).

The energy of interaction of two atoms at a distance $r$ can be described [58] by the Lennard-Jones potential $U_{\rm LJ}(r, r_0) = \varepsilon\left[\left(\frac{r_0}{r}\right)^{12} - 2\left(\frac{r_0}{r}\right)^{6}\right]$, where $r_0$ is the optimal distance between atoms, and $\varepsilon$ is their interaction energy at this distance. Thus, $U_{\rm LJ}(r_0+\delta r, r_0) + U_{\rm LJ}(r_0+\delta r, r_0)$, the potential energy of the central atom located at a distance of $r_0+\delta r$ from one of them and $r_0-\delta r$ from the other, is

$$U(\delta r) = 2U_{\mathrm{LJ}}(r_0, r_0) + \Delta U_{\mathrm{LJ}}(r_0, \delta r),$$

where $U_{\mathrm{LJ}}(r_0, r_0) = 2\varepsilon$ does not depend on the displacement $\delta r$, and $\Delta U_{\mathrm{LJ}}(r_0, \delta r) = \varepsilon\left[72\left(\frac{\delta r}{r_0}\right)^2 + 1722\left(\frac{\delta r}{r_0}\right)^4 + \cdots\right]$ is the displacement energy. Replacing $\frac{\delta r}{r_0/12}$ with $x$, we see that this $\Delta U_{\mathrm{LJ}}(r_0, \delta r)$ is proportional to $(x^2 + x^4/6)/2 + \ldots$ .

So, in the case of "a particle located between two other particles", the sharper (than in a harmonic potential $U(x) \propto x^2/2$) increase of $\Delta U_{\mathrm{LJ}}(x)$ with the deviation $x$ from the equilibrium should lead to a Mpemba-like effect connected with an ordinary atomic interaction.

Thus, two factors are required for the deterministic Mpemba effect to occur in an oscillator. These are: inertia—"kinetic memory" (so that kinetic energy acts as a "hidden ordering parameter" [60] alongside potential energy, the explicit ordering parameter)—and a sharper than a quadratic increase in potential $U(x)$ upon deviation $x$ from equilibrium, because, in the absence of friction, the law of conservation of energy reads that $\frac{m}{2}\left(\frac{dx}{dt}\right)^2 + U(x) = U(X_0)$.
